# Non-cross-linked polystyrene-supported 2-imidazolidinone chiral auxiliary: synthesis and application in asymmetric alkylation reactions

**DOI:** 10.3762/bjoc.9.248

**Published:** 2013-10-15

**Authors:** Quynh Pham Bao Nguyen, Taek Hyeon Kim

**Affiliations:** 1 School of Applied Chemistry and Center for Functional Nano Fine Chemicals, Chonnam National University, Gwangju 500-757, Republic of Korea

**Keywords:** asymmetric alkylation, chiral auxiliaries, chiral carboxylic acids, 2-imidazolidinone, non-cross-linked polystyrene

## Abstract

Asymmetric alkylation reactions using non-cross-linked polystyrene (NCPS)-supported 2-imidazolidinone chiral auxiliaries were successfully investigated with excellent diastereocontrol (>99% de). The recovery and the recycling of this soluble polymer-supported chiral auxiliary were achieved in order to produce highly optical pure carboxylic acids.

## Introduction

Chiral auxiliaries have been proven as a powerful tool for the asymmetric synthesis of highly optical pure compounds used in pharmaceuticals or agrochemicals [1–2]. The recycling and reuse of expensive chiral auxiliaries is a challenge in organic synthesis and remains under-developed [3]. In this field, polymer-supported synthesis has emerged as a versatile technique, which involves the attachment of chiral auxiliaries to the polymer carriers for convenient purification, recovery and reuse. Most recently, cross-linked, insoluble polymer supports, such as Merrifield and Wang resins, have been mainly explored [3–5]. However, the unavoidable heterogeneous-phase reaction and the spectroscopic analysis of the functionally supported products are extremely problematic. In an attempt to bridge the gap between the solid phase and solution phase synthesis, non-cross-linked soluble polymer supports have attracted great interest because of some advantages such as high reactivity, easy analysis and purification of products [6–12].

Asymmetric alkylation reactions using polymer-supported chiral auxiliaries have not been widely investigated yet [13–26]. In this field, solid supported chiral auxiliaries such as Evans' 2-oxazolidinones [13–15,17] and pseudoephedrines [16,22] have been mainly explored, however, a high stereocontrol has still not been accomplished (55–97% ee). As mentioned above, although very attractive from the viewpoint of synthesis, soluble polymers have received little interest as supports for chiral auxiliaries in asymmetric alkylations. The literature contains only one report by Yang et al. about non-cross-linked polystyrene (NCPS)-supported 2-phenylimino-2-oxazolidine, which facilitated the synthesis of several chiral amides in excellent stereoselectivity (>96% ee). However, the sterically undemanding methylation has not been investigated [20]. Recently, we introduced 2-imidazolidinone [26–27], a versatile auxiliary for asymmetric synthesis, into the solid support [18–19]. As a part of our ongoing research, we herein report a novel NCPS-supported 2-imidazolidinone chiral auxiliary that exhibited excellent diastereocontrol. Its recycling and reuse for the synthesis of several chiral carboxylic acids are addressed.

## Results and Discussion

**Scheme 1:**
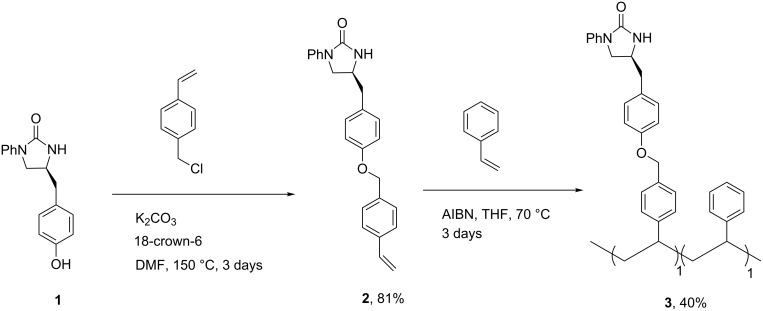
Synthesis of NCPS-supported 2-imidazolidinone chiral auxiliary **3** through direct copolymerization of 2-imidazolidinone derived monomer **2** with styrene.

First, the 2-imidazolidinone chiral auxiliary **1** was prepared in the solution phase from the commercially available *O*-benzyl-L-tyrosine in four steps, as previously reported [18]. For the synthesis of our envisioned homogeneous polymer, we began to directly co-polymerize a pair of functionalized 2-imidazolidinone-derived monomers **2** with styrene in a 1:1 ratio using AIBN as a radical initiator in THF (Scheme 1) [9]. After the precipitation in cold methanol, the expected polymer **3**, which was soluble in THF, CH_2_Cl_2_, and DMF, was obtained in low yield (40%).

**Scheme 2:**
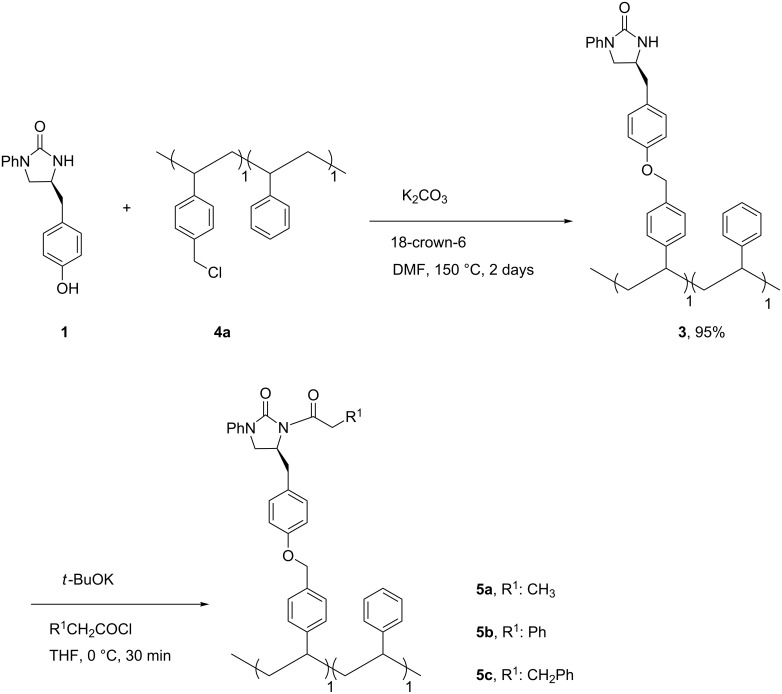
Synthesis of NCPS-supported *N*-acylated 2-imidazolidinone chiral auxiliaries **5**.

Therefore, to save our 2-imidazolidinone chiral auxiliary, an alternative approach, in which the starting soluble polymer **4** was first prepared and then linked to 2-imidazolidinone chiral auxiliary **1**, was examined (Table 1 and Scheme 2) [6,12]. In an attempt to optimize the amount of loading sites in the soluble polymer and its solubility, several non-crosslinked chloromethylated polystyrenes **4** were simply synthesized according to reported procedures [6,8] in different feed ratios of 4-chloromethylstyrene and styrene of 1:1, 2:1, and 1:0 (Table 1). The NCPS **4a**, **4b**, and **4c** were obtained in 46, 40, and 52% yield, respectively, and showed also good solubility in THF, CH_2_Cl_2_, DMF, and benzene, and insolubility in methanol and water. Unlike the solid supports [13–26], the soluble NCPS polymers **4** were characterized easily by the ^1^H NMR spectra, which were in accordance with the proposed structures. The immobilization of the 2-imidazolidinone chiral auxiliary **1** onto NCPS **4a** by using K_2_CO_3_ and 18-crown-6 in DMF [12], to our delight, gave the corresponding polymer **3** in 95% recovery mass balance yield (Scheme 2). The ^1^H NMR of polymer **3** (Supporting Information File 1) revealed its complete attachment with a loading level of 2.05 mmol/g. Our effort to introduce 2-imidazolidinone chiral auxiliary **1** into NCPS **4b** to increase the loading capacity of the NCPS-supported 2-imidazolidinone chiral auxiliary was unsuccessful because the obtained polymers were insoluble in any organic solvents. As a result, polymer **3** with 50% active sites was balanced between the largest number of reactive sites and the most suitable solubility property of the polymer itself. *N*-Acylation of polymer **3** proceeded smoothly to yield polymers **5** in quantitative yields, as evidenced from the ^1^H NMR spectra of polymers **5** (Supporting Information File 1). Polymers **5** were also highly soluble in THF, CH_2_Cl_2_, and DMF and insoluble in methanol and water, which allowed the implementation of solvent extraction techniques commonly used in classical organic synthesis.

**Table 1:** Synthesis of non-crosslinked chloromethylated polystyrenes **4**.

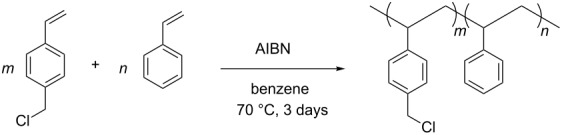							

entry	feed ratio (*m*/*n*)	polymer	yield^a^ (%)	loading (mmol/g)	*M*_n_^b^ (dalton)	*M*_w_^b^ (dalton)	PD^b^

1	1/1	**4a**	46	3.89	3,180	5,992	1.9
2	2/1	**4b**	40	4.89	4,436	7,370	1.7
3	1/0^c^	**4c**	52	6.55	8,604	11,817	1.4

^a^Calculated on the base of the monomer feed ratio. ^b^Determined by GPC relative to polystyrene standards. ^c^Homopolymer.

Next, asymmetric benzylation, as a model alkylation reaction with the soluble polymer **5a,** was examined for optimization of the reaction conditions (Table 2). NaHMDS was the best base, as compared to LiHMDS and LDA, to obtain the product **7a** in good yield and excellent diastereoselectivity (Table 2, entry 4). While the ^1^H NMR spectrum of soluble polymer **6a** lacked the necessary clarity to examine the alkylation process, another cleavable linker strategy was applied by treating the alkylated polymer **6a** with trifluoroacetic acid (TFA) for 5 min at room temperature to produce compound **7a**, which could be conveniently monitored by classical thin layer chromatography (TLC) [18]. Additionally, in the solid phase asymmetric alkylation reactions, in which Evans' 2-oxazolidinone chiral auxiliaries were used, the yield and stereoselectivity were too dependent on the base, reaction time, and supported resins. As an example, the base-catalyzed epimerization of the chiral center was responsible for the poor de value [17], and Merrifield resin was an unsuitable support [14]. In contrast, the asymmetric benzylation in the soluble polymer **5a** gave excellent diastereoselectivity (>99% de) which was not affected by the base or reaction time (Table 2, entries 2–5). Interestingly, although having a similar functional structure to that of the Merrifield resin, soluble polymer **4a** showed the potential to be a good support under our condition.

**Table 2:** Optimization of asymmetric benzylation reaction.

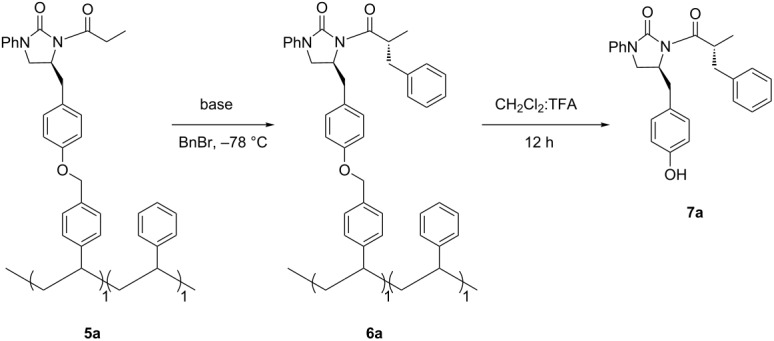					

entry	base	equiv of base/BnBr	time (h)	yield^a^ (%)	de^b^ (%)

1	LiHMDS	3/10	24	trace	—
2	LDA	3/10	24	25	>99
3	NaHMDS	3/10	24	32	>99
4	NaHMDS	5/10	24	40	>99
5	NaHMDS	5/10	12	30	>99

^a^Yield of **7a** after four steps based on the loading of NCPS **4a**. ^b^Determined by ^1^H NMR and HPLC (chiralcel ODH column).

With the optimized reaction conditions in hand, the asymmetric alkylation reactions using NCPS-supported 2-imidazolidinone chiral auxiliaries **5** were investigated. As shown in Table 3, benzyl bromide, allyl iodide and methyl iodide reacted very well to give the alkylated products **7** in moderate to good yields and excellent de values of >99% (Supporting Information File 1). Especially, the diastereocontrol of asymmetric methylation in this case was better than that in the solution phase (89% de) [27] due to the sterical hindrance of the polymeric support. While the most extensively explored Evans' 2-oxazolidinone chiral auxiliary required a complicated linker [15] and solid supports [14], as well as special treatment, in which the excess base responsible for epimerization was removed before adding the alkylated reagents [17], to obtain the high stereoselectivity control, the NCPS-supported 2-imidazolidinone chiral auxiliary **3** was simply prepared and asymmetric alkylations of it proceeded smoothly to produce the products in excellent diastereoselectivity (>99% de) without using any special strategy. To the best of our knowledge, no studies have reported successful diastereocontrol by using other polymer-supported chiral auxiliaries in asymmetric alkylation reactions, especially in the methylation case [13–26]. Therefore, 2-imidazolidinone **1** could be regarded as a powerful chiral auxiliary for asymmetric alkylations in polymer supports.

**Table 3:** Diastereoselective alkylations before TFA cleavage.

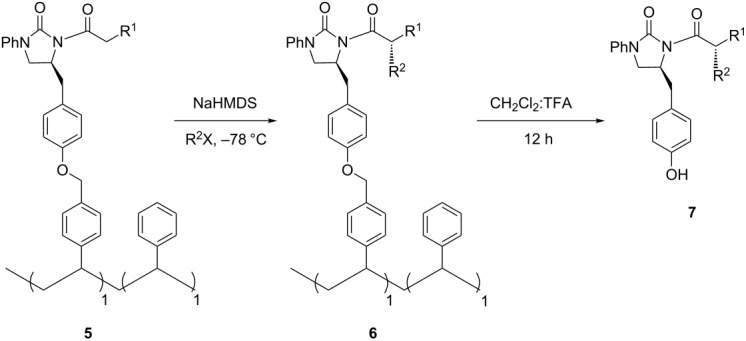							

entry	R^1^	substrate	R^2^X	time (h)	product	yield^a^ (%)	de^b^ (%)

1	CH_3_	**5a**	BnBr	24	**6a**, **7a**	40	>99
2	Ph	**5b**	BnBr	24	**6b**, **7b**	34	>99
3	Bn	**5c**	CH_2_=CHCH_2_I	24	**6c**, **7c**	36	>99
4	Bn	**5c**	CH_3_I	36	**6d**, **7d**	27	>99

^a^Yield of **7** after four steps based on the loading of NCPS **4a**. ^b^Determined by ^1^H NMR and HPLC (chiralcel ODH column).

For the removal of the NCPS-supported 2-imidazolidinone chiral auxiliary, the alkylated polymer **6a** was treated with NaOH to produce the chiral acid **8a** in excellent de value of >99% (Table 4, entry 1). The highly recovered yield of polymer **3** (>90%) and the similarity of its ^1^H NMR spectrum to that of the fresh prepared one encouraged us to study its recycling. As expected, our NCPS-supported chiral auxiliary **3** could be reused three times to produce the chiral acids **8a**, **8b**, and **8c** in excellent ee values (Table 4, entries 1–3). To our surprise, although the asymmetric methylation proceeded very well with excellent diastereocontrol (>99 de), epimerization occurred under NaOH cleavage condition. Consequently, chiral acid **8d** was obtained in only 89% ee (Table 4, entry 4).

**Table 4:** Synthesis of chiral acids **8** and recycling of polymer **3**.

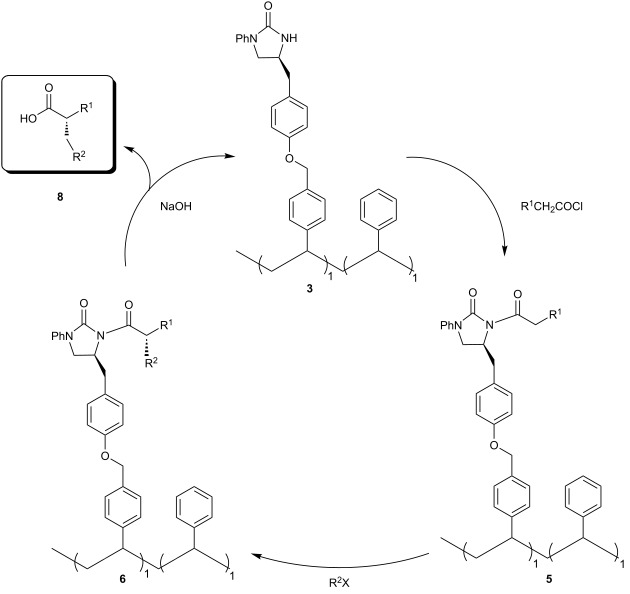					

run	R^1^	R^2^X	product	yield^a^ (%)	ee^b^ (%)

1^st^	Me	BnBr	**8a**	55	99
2^nd^	Ph	BnBr	**8b**	43	99
3^rd^	Bn	CH_2_=CHCH_2_I	**8c**	46	99
4^th^	Bn	CH_3_I	**8d**	38	89^c^

^a^Yield of **8** after four steps based on the loading of NCPS **4a**. ^b^Determined by HPLC (chiralcel ODH column) after conversion of chiral acids **8** to the corresponding chiral esters using trimethylsilyldiazomethane. ^c^de of **7d** >99% obtained from the recovered polymer **3**. The ee of **8d** obtained from the freshly prepared polymer **3** was the same as that of the recovered polymer **3**.

## Conclusion

In summary, an NCPS-supported 2-imidazolidinone chiral auxiliary was developed with the following three clear advantages: (1) high loading capacity (more than 1.0–1.5 mmol/g loading of Wang resin or Merrifield resin), (2) remarkable solubility properties that are extremely useful for reaction and workup conditions, and (3) functional group content that can be readily quantified by simple ^1^H NMR analysis. In addition, the asymmetric alkylation reactions using NCPS-supported 2-imidazolidinone chiral auxiliary were successfully investigated with excellent diastereocontrol (>99% de). In almost cases, the recovery and recycling of this soluble polymer-supported chiral auxiliary were achieved to produce highly optical pure carboxylic acids (99% ee). The alkylated polymer **6a** was also treated with LiAlH_4_ and NaOMe. Unexpectedly, racemization occurred under these cleavage conditions to produce the corresponding chiral alcohol and ester in 80% and 77% ee, respectively.

## Experimental

**Typical procedure for *N*-acylated reactions in NCPS-supported 2-imidazolidinone chiral auxiliary 3**: The polymer **3** (1 equiv) was dissolved in THF under argon atmosphere at 0 °C. Then, a 1 M solution of *t*-BuOK in THF (5 equiv) was added dropwise, followed by the addition of acyl chlorides (10 equiv). The reaction mixtures were stirred for 30 min, quenched by adding saturated NH_4_Cl solution and extracted with CH_2_Cl_2_. The organic layers were evaporated and the obtained viscous solutions were dropped into cold methanol. The precipitated solids were filtered, washed with water and methanol, and dried at 60 °C under vacuum to yield the polymers **5**.

**Typical procedure for asymmetric alkylation reactions using NCPS-supported 2-imidazolidinone chiral auxiliaries 5***:* Under Ar atmosphere, the polymers **5** (1 equiv) were dissolved in THF and cooled to −78 °C, following by the dropwise addition of 1 M NaHMDS (5 equiv). After continuously stirring for 2 h at the same temperature, the alkyl halides (10 equiv) were added and allowed to react for 24–36 h. Then, the reaction mixtures were quenched with saturated aqueous NH_4_Cl and extracted with CH_2_Cl_2_. The organic layers were evaporated and the obtained viscous solutions were dropped into cold methanol. The precipitated solids were filtered, washed with water and methanol, and dried at 60 °C under vacuum to yield the polymers **6**.

**To monitor the reactions**: A small amount of polymers **6** (ca. 3–5 mg) were treated with an excess 1:1 v/v mixture of CH_2_Cl_2_ and TFA (5 mL) for 5 min. Then, the reaction mixtures were evaporated under vacuum to produce the alkylated products **7,** which were conveniently monitored by TLC.

**Cleavage reactions:** The alkylated polymers **6** were treated with an excess NaOH 2 N: dioxane (1:1 v/v). After 2 h at 100 °C, the reaction mixtures were extracted with CH_2_Cl_2_. The organic layers were evaporated and the obtained viscous solutions were dropped into cold methanol. The precipitated solid was filtered, washed with water and methanol, and dried at 60 °C under vacuum to yield the recovered polymer **3**. The water layers were acidified to pH 2 with HCl and extracted with ethyl acetate. The crude product was purified by flash column chromatography to yield the acids **8**. The alkylated polymer **6a** was also treated with LiAlH_4_ and NaOMe. Unexpectedly, racemization occurred under these cleavage conditions to produce the corresponding chiral alcohol and ester in 80% and 77% ee, respectively.

## Supporting Information

File 1 ^1^H NMR spectra of **3**, **5a**, **6a**, **7a–d**, and HPLC data of **7a–d**.
