# Supplementary material for: Non-cross-linked polystyrene-supported 2-imidazolidinone chiral auxiliary: synthesis and application in asymmetric alkylation reactions
Source: Beilstein J Org Chem. 2013 Oct 15;9:2113–9. doi: 10.3762/bjoc.9.248 (PMC3817508; doi:10.3762/bjoc.9.248)
Supplement: File 1 — 1H NMR spectra of 3, 5a, 6a, 7a–d, and HPLC data of 7a–d. [file Beilstein_J_Org_Chem-09-2113-s001.pdf]

# Supporting Information

for

# Non-cross-linked polystyrene-supported 2-imidazolidinone chiral auxiliary: synthesis and application in asymmetric alkylation reactions

Quynh Pham Bao Nguyen and Taek Hyeon Kim*[§]

Address: School of Applied Chemistry and Center for Functional Nano Fine Chemicals, Chonnam National University, Gwangju 500-757, Republic of Korea

Email: Taek Hyeon Kim* - thkim@chonnam.ac.kr

* Corresponding author

[§] Tel: +82 62 530 1891; Fax: +82 62 530 1889

## $^{1}$H NMR spectra of 3, 5a, 6a, 7a–d, and HPLC data of 7a–d

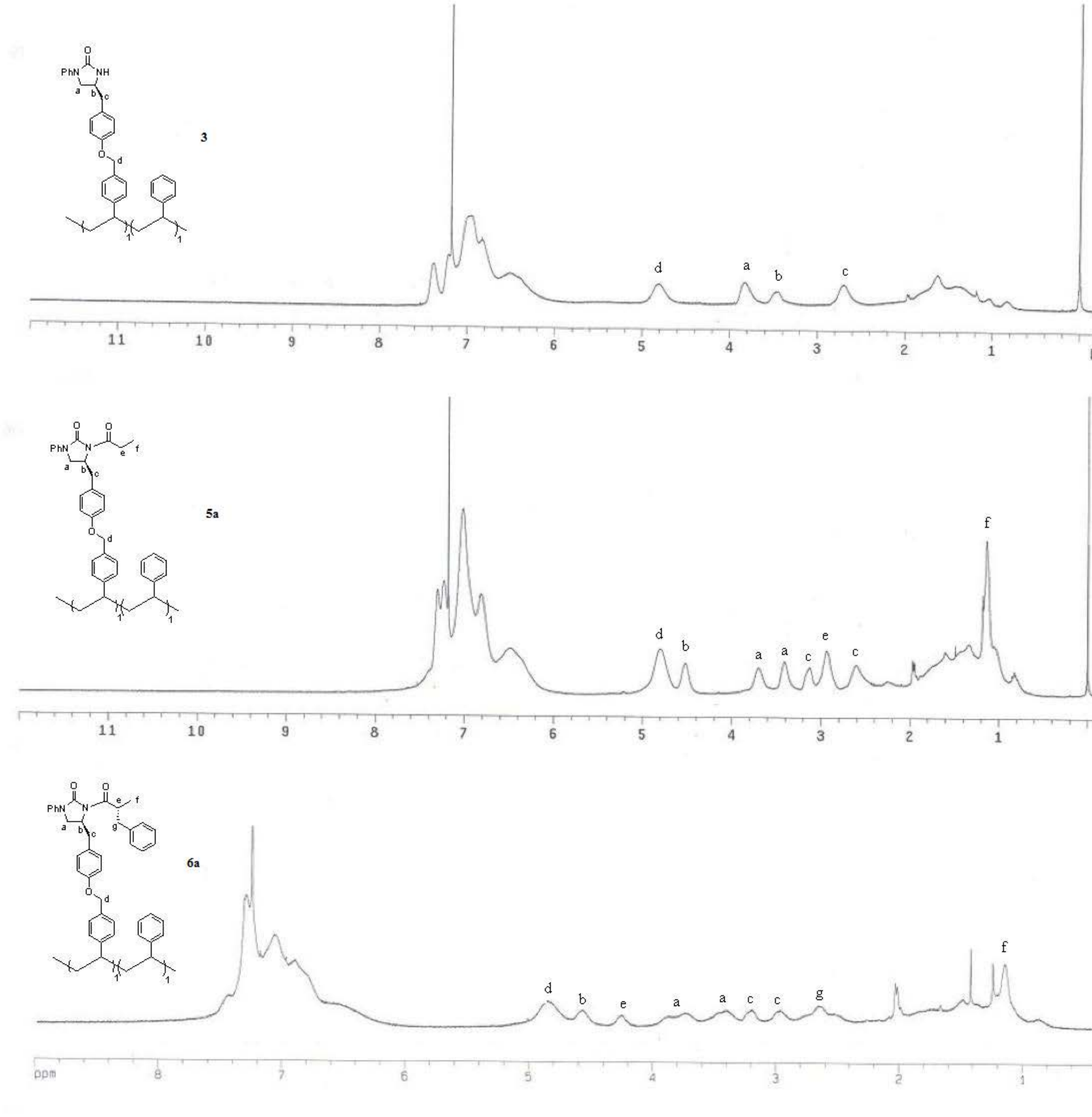

**Figure S1:** $^{1}$H NMR spectra (300 MHz, $CDCl_3$) of polymers **3**, **5a** and **6a**.

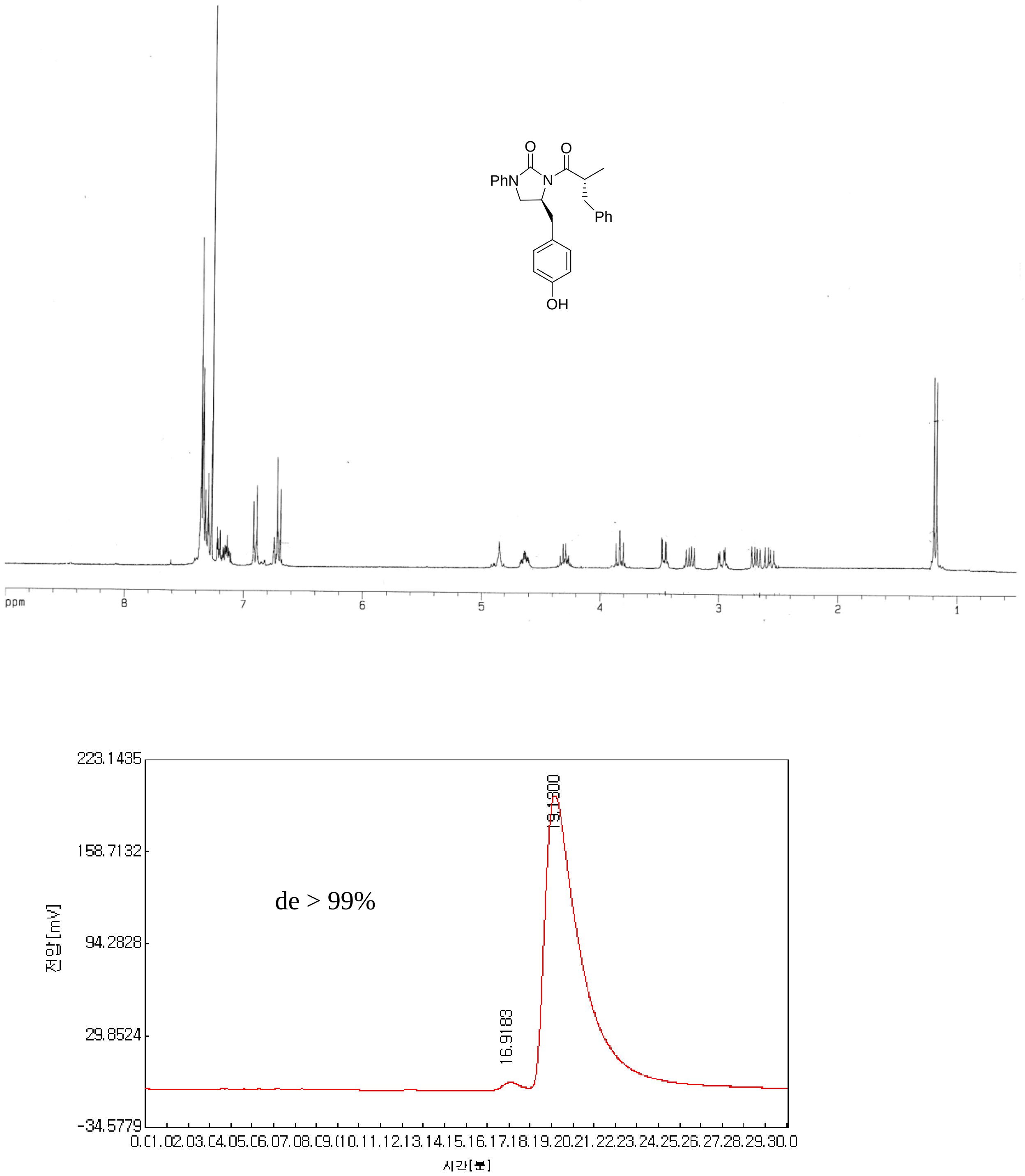


**Figure S2:** $^{1}$H NMR and HPLC data of compound **7a**.

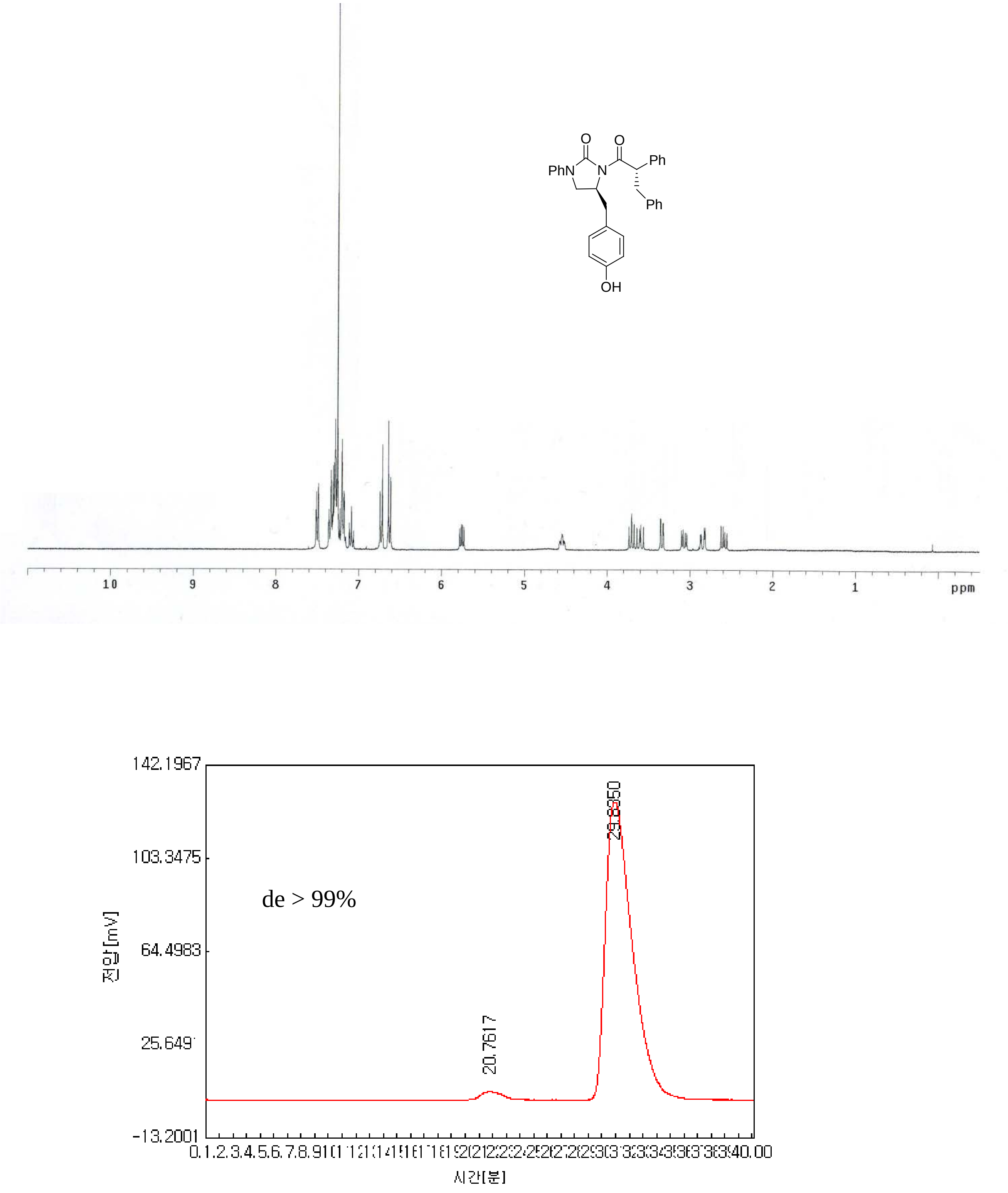


**Figure S3:** $^{1}$H NMR and HPLC data of compound **7b**.

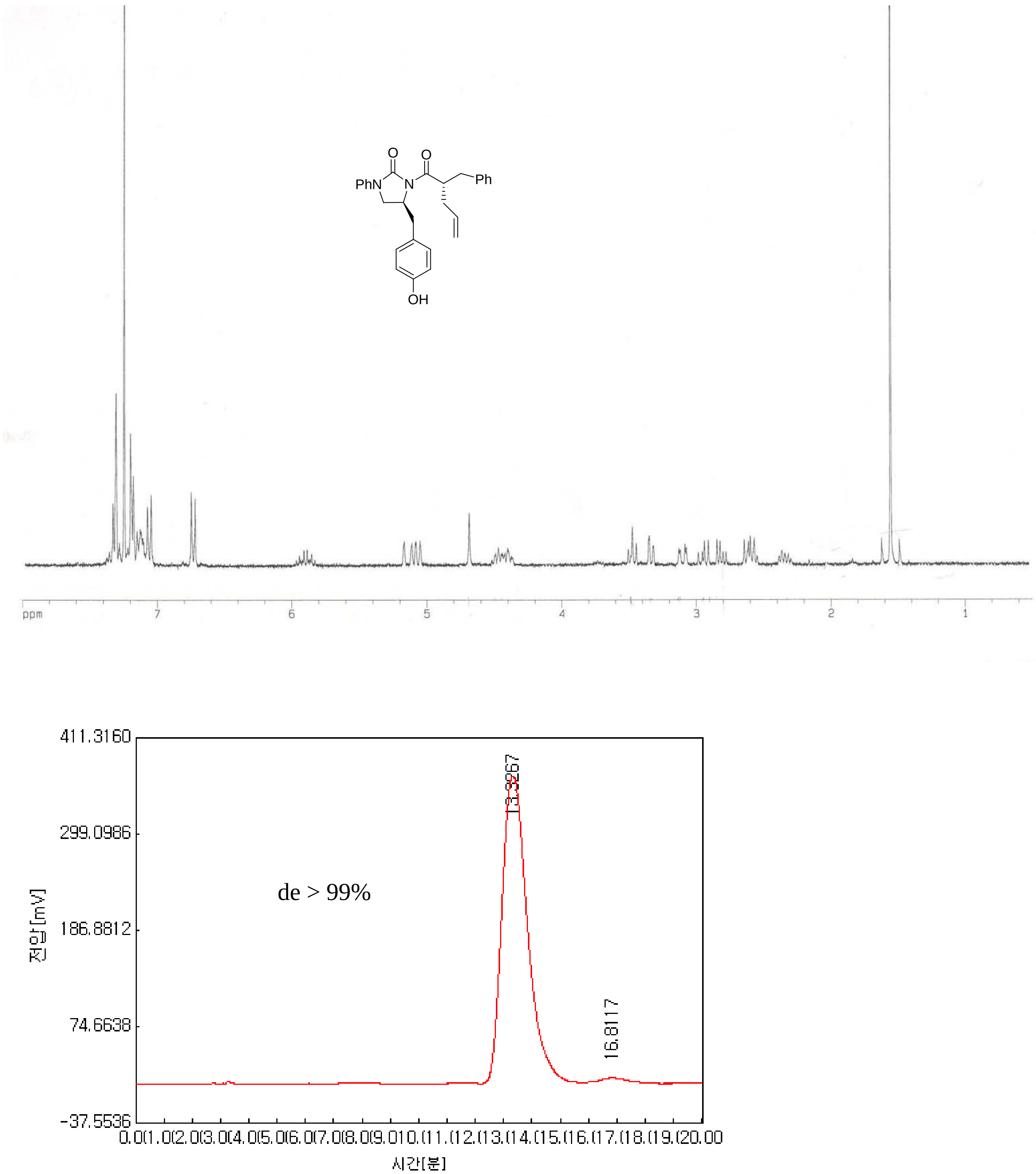


**Figure S4:** $^{1}$H NMR and HPLC data of compound **7c**.

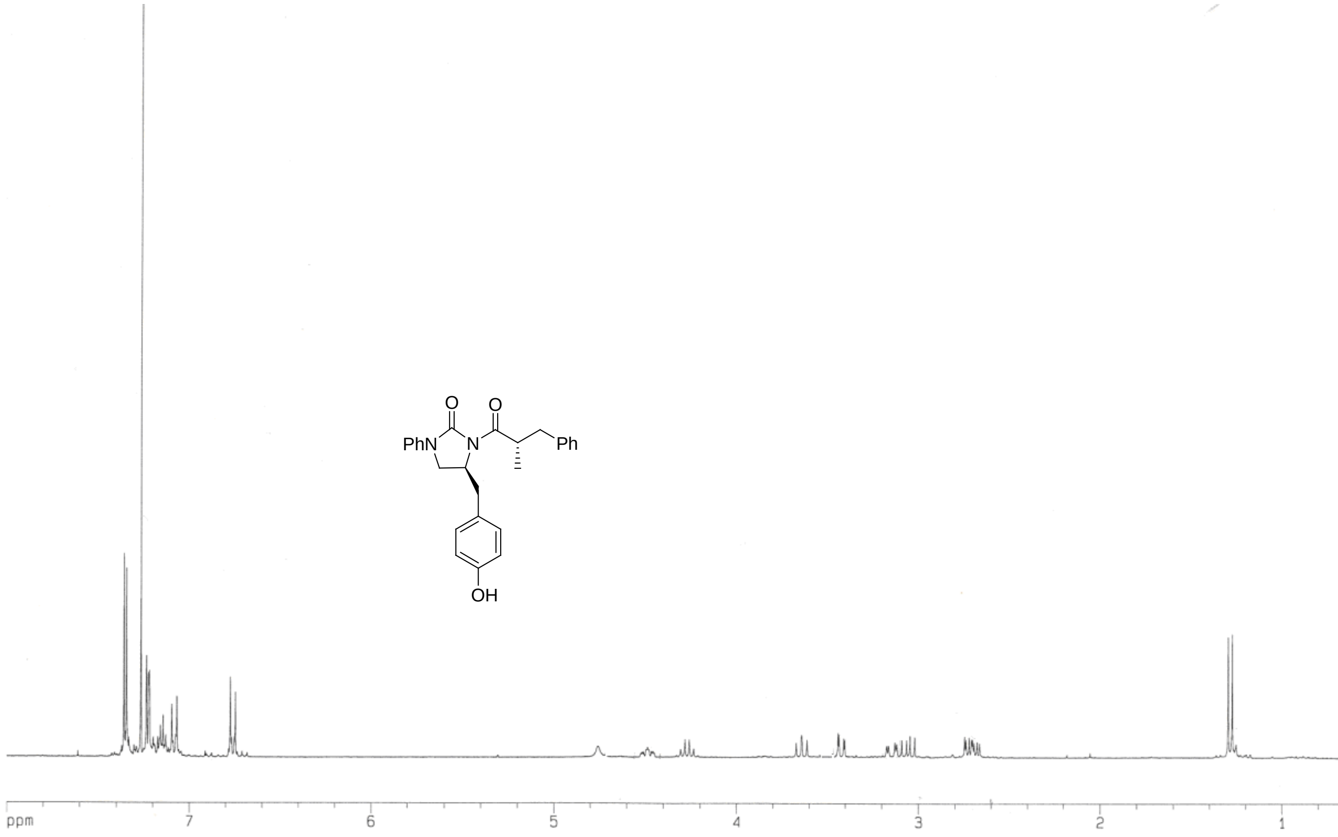


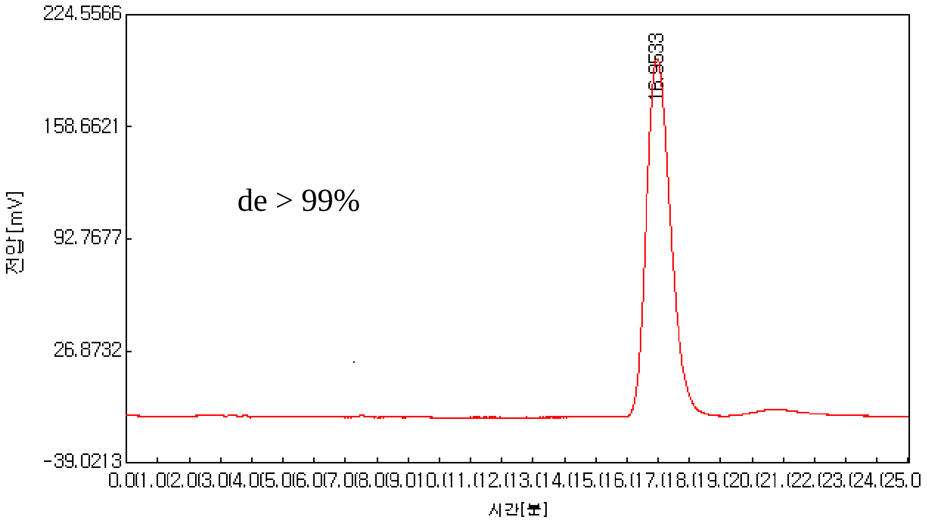


**Figure S5:** $^1$H NMR and HPLC data of compound **7d**.
